# Medical Fogieism

**Published:** 1854-11

**Authors:** 


### MEDICAL FOGIE-ISM.

This term has been so often used, so frequently quoted, and so strongly emphasised, that we are led to suppose there is opprobrium in its meaning. In all respect to those with whom it is in such familiar use we ask most beseechingly what it is? Is it intended to apply to those who have been long in the service and have laid up stores of knowledge by observation, experience and research? Is it meant for those whose heads time has silvered with grey hairs, and furrowed deep fissures of anxious care in their faces? Is it used to scout from the profession all such, and heap odium upon their heads after a long, eventful and useful life? Much as we admire the energy, activity and emulation of the young in their march of progress, and much as we desire to see them imbued with a just ambition for greater usefulness, we must be allowed to enter our solemn caveat to this tirade against age and its concomitant—*experience*. We venerate grey hairs and we respect age, and in spite of such young aspirants as would cast a slur upon experience and years, we will *continue* to venerate and respect them, and so will the world. We protest against such a spirit as would *elbow*

out of the way the feeble, tottering, and time honored deciple of medicine who has manfully kept his place in the ranks, and still occupies his position in the "fore front," doing battle with that invincible daring as if it were a sacred duty, and with a zeal inspired by humanity. It is sickening to hear those who have but a short time since escaped from the "apron-strings" of their *alma mater* jeer at what they are pleased to call *foibles* and *trifles* incident to age and long years of experience. And who has not observed how painful it is for some to strain out an expression of commendation upon those much older than themselves, as if even a word favorably spoken cost a parturient effort. It is enough for the old to feel the embarrassments of enfeebled age, without having contempt heaped upon their physical decline. It is an insult to nature herself, and is a self-condemnation in advance, for as certain as that there is a law of gravitation, there is also a law of decadence which will just as surely overtake all, and yet they effect to despise it. Look at the effect of time and years upon the form of the justly distinguished Mott. Will "young physic" dare to cast contumely upon the man of whom the American profession may well be proud. Why is his counsel sought for by even those who are daily crying out "fogie-ism?" Then there is the little attenuated and bowed-down physical frame of Mussy who has scarcely body large enough for his great soul, is he to be derided as an "old Fogie?" If so, why is he listened to with so much attention, and why are his opinions so much respected? Is there one who dare raise his voice of excommunication against these venerated and venerable fathers with many others equally distingushed? If there be, it were better for *his* position and credit had he held his peace. The same may be said of others whom we could name, whose prominence is acknowledged and whose merits will bring their reward in the abundant gratitude of the profession. Let those who deride, but imitate their virtues and emulate their useful lives, and even then these scoffers will fall short of the high mark to which these sires have attained. Let them reflect that it is but human nature to err, and that although the old may cling too long and too tenaciously to erroneous views, yet the young have their vanities, foibles and conceits. After all, there is much to be learned by experience alone; therefore, the veterans in the service can best lead in progress, and are the true conservators—the first, the safest and most efficient reformers. We hope to hear no more taunts or revilings toward those who are traveling downward toward the tomb, which will ultimately bury more mental power, talent, and usefulness, than such sneering revilers could ever conceive of, or attain to, should their thread of existence be spun out the length of that of Methusaleh. Time on earth would not be long enough for them to reach to such a goal.

There is a wide difference between a ripe experience and embecile dotage—just as much as there is between infancy and maturity.—It is a misfortune with some, however, never to reach a maturity, but rather their infancy is merged into mental superanuation. Such is the case with those who are "*wise in their own conceits*," and under the accumulated weight of vanity and self-importance, of arrogance and self-adulation, they sink down, remain down, and amid their sepulchal murmurings and mutterings, the echo of the slang of "fogie-ism" is heard reverberating in bitter and vindictive tone.

We are the friends of progress—our position and efforts are unmistakable evidences of it, without presuming too far, allow us to say that if our shoulders are not to the wheels of the car, in all modesty we would ask, whose are? Our strength may not be *herculian*, but our will is good, and if health and life are to be ours within the ordinary allotment, we will continue our best efforts.—But we desire to see no obstructions thrown in the way, nor discouragements thrown upon those who have labored and who still continue to work. We are disposed to respect the opinions of those who have enjoyed the benefits of a long experience, and if they differ with others in the choice of the most effective mode of action, let them be gently and affectionately advised and counseled. Let them not be jeered at—railed at—*scoffed at.*

We would at the same time say to those who are of the sect of "*young physic*," to those in particular whose energy and talent enable them to bound forward on the high-way of progress, to persevere in their efforts, for in the future before them there is in reservation a fame as brilliant and dazzling as that which now gilds the sunset of those soon to give place to them. The legacy which these last will bequeath will be as responsible as the trust will be sacred. But to prove worthy of it, they must be respectful and liberal.—

They must not pronounce every opinion a *dogma* simply because it has been avowed by one whose head is variegated by a few gray hairs. It is impossible for the mind to discard opinions based upon what it conceives and judges to have been facts developed from time to time in the daily discharge of duty and in the progress of observation ; and yet it may happen that the data *may* betray and the premises *may* prove false and erroneous. Nor should the old and experienced frown discouragement upon those younger than themselves because they advance new and different views, *simply* and *solely* because they emanate from the young. This will bring upon them contempt, and disdain, and dislike will be engendered.—It will prove suicidal to the medical profession to originate parties or sects in the body of the profession, for we should present an unbroken front, in order the better to contend against the empiricism of the country whose life-blood will be drawn from intestine divisions and feuds among ourselves.

In conclusion we will introduce one of those who has been met on his onward way with the salutation, "Go up thou bald head," whose distinguished services deserve the heart-warm thanks of the medical men, not of this country only, but those "over the waters." Let him speak for himself, for surely he is "of age." After all his chief sin lies in the manner and not in the matter of his works. He is an original "thinker," and if he is somewhat original in the use of the representatives of his thoughts, it is a small fault and should be reformed by respectful kindness and not by reproach.

### PROFESSOR MEIGS' REPLY TO CRITICS.

*Philadelphia*, *May* 6, 1854.

DEAR SIR:—As it is common to all men to prefer commendation rather than reproof, I could not well avoid a feeling of regret, not unmixed with surprise, on receiving your note of the 1st inst., which was marked "confidential." I regret, in the first place, that your journal should be the medium by which I am to be assailed, and I was surprised to find that you should use it against a work of mine with which you are apparently not dissatisfied. At least, I gather from the tenor of your letter that you do not disapprove of the tract in question.

I am much obliged to you, sir, for the favorable expressions and kind wishes contained in the closing paragraph of your letter, and beg to assure you, you are quite correct in supposing that I "make a better use of 'my' time than those who read reviews of themselves after having written the best books extant." I believe there are not a few reviews of my publications that I have not read; and while it is true that I should thankfully receive and strive to improve every truly obvious suggestion in the way of emendation, I confess I have but very little concern in the opinions of angry and unreasonable or incompetent writers of criticism, some of which I have found to be beneath contempt for knowledge or temper exhibited by them.

I hope that I have not, by any one, been charged with the indecency of praising my own writings, which have often been the subjects of very sharp comment. I know and admit that my writings have many faults; but I claim that even were I a good writer, I have been too busy a man to write with care, or with very special regard to the manner of expressing my thoughts. If I should have waited for time to write, I should never have made public a line on topics; and yet, as you know, I have written a good deal: perhaps I should have been a wiser man if I had never published a paragraph on medicine! and were I governed only by the opinions of these young gentlemen of our brotherhood, who *do* most of the American medical reviews, I should long ago have resolved never thereafter to open my mouth in their presence, but, holding my peace, leave them alone in their self-sufficiency.

It is a difficult thing for a man to judge on a question of this kind. Here now are young people in New York and Virginia, and elsewhere, who review not my books only, but me, even when my books are not in the caption; and who inform the public that I cannot write English, and that what I do say is wholly unintelligible, and, worse and worse, that what I have written is "unworthy of his (my) eminent position."

I have not claimed to be in an eminent position, saving and excepting only, that I shall ever deem it a fortunate and creditable circumstance that I am sustained by my colleagues of the College, conjointly with whom I have labored as a public instructor of students of medicine, in perfect harmony and concord, for a great many years. This, I presume I may, without vanity, be allowed to regard as an enviable position, seeing that our medical brethren in the States do send to us a great number of their pupils, which is a certain mark of their confidence and respect.

I know not, then, what these young gentlemen mean by "his eminent position," unless they be pleased to refer to my writings, which nevertheless they do reprobate, and, I might say, truculently condemn and destroy—if they be, indeed, destroyed by these public spirited and most learned guardians of our sacred fane!

What would you have me to do, Dr. Dowler? Shall a man lay his hand on his mouth and his mouth in the dust, because a * * * writer of squibs shall deem him unworthy of his "eminent position?"

I do think that Heaven knows I never wrote for my own sake, but for the sake of my brethren, to whom I owe an unpayable debt of thanks and grateful respect for their goodness, by me scarcely deserved. I say that I am deeply in debt to my medical countrymen for the some thousands of their students whom they have permitted to hear my public lectures, and for their approbation of my writings, most clearly expressed in the fact that they have taken 15 or 20,000 volumes of them from my booksellers, and are now asking me for others that I am preparing to send them. In fact, I have just finished for the binders a new and enlarged edition of my Letters on Woman, which I hope may be found emended as well as augmented, for it was much abused, with the rest.

I repeat, then, what ought I to do? Am I to believe the young gentlemen, the sophomore *scollards* [?] who assail me, or may I not venture rather to rely on the seniors, my brethren, who buy 20,000 volumes of my medical tracts, and ask me for others that are coming? I have too good an opinion of American doctors to think they would purchase so large a library that has in it neither English nor common sense.

As to the particular tract which you tell me is to be reviewed in your forthcoming number, I will be so weak as to confess, I should be sorry to find it failure, not on account of the personal mortification merely, but because I have good reasons to believe it contains much sound and wholesale instruction, well fitted to aid the young and inexperienced brethren in a difficult department of clinics; wherein many, nay the majority of us, commit the most scandalous blunders, and do the most blameable malpractice.

I hope I have not the least desire to rescue the volume, however, much I confide in the principles and methods which I have inculcated, from a condign condemnation. Yet I confess it is hard for me to understand how it should be, that, while supposing myself to be very intimately acquainted with the history and bibliography of that particular subject, I should make the grave mistake of regarding the book as not only a useful but an original and novel exposition of these matters, if it should in the end prove to be not worth a rush. Assuredly, considering the place I have long occupied as a practitioner and teacher, the duty I owed to my brethren of being a man of studious habits, as well as a careful observer of diseases and results of treatment, I ought by this time to have learned something worthy of being told to others. Still, your reviewer may be a person far more variously and accurately informed than I, and so prove himself quite able to show that I have learned nothing in cases that have attracted much of my attention for many years.—Let him, in that case, cut my book into shreds if he will; I shall endeavor to think no evil of him on account of his evil intent towards me, or my book rather. If he rails at us, much happiness and self-gratulation may he find in his railing. I shall endeavor to find contentment, nevertheless, and to that end perhaps I might do well to read in the Bible. In the 2d chapter of II Book of Kings, I shall find a story concerning the prophet Elisha; he was old and well strichen in years, and so am I; he had a bald head, and so have I; he went on his way in the world, and so do I; he met angry and naughty boys, so have I; they scorned his gray hairs and hooted at his bald crown; probably they thought him unfit for his "eminent station," and they cried out upon him, "Go up, thou bald head; go up, thou bald head." The prophet turned and "cursed them," so do not I; and the Lord sent two she-bears out of the mountain, and "they tare forty-and-two of those children that day." I am very sorry for the poor dear little Jew boys that were torn, and I hope my reviewers may keep clear of all such, and other vermin. And I even go so far in humanity as to trust, humbly, they will not feel themselves hurt by the reflection that their brethren and mine have bought some 20,000 volumes of medical works from a writer whom they so greatly disapprove.

I heartily reciprocate your kind wishes for my welfare; and, while I regret you should use your journal to do me hurt and damage, I am not the less an admirer of your talents and industry, and I rest with respectful consideration.

Your Servant, CH. D. MEIGS.

Dr. B. DOWLER, New Orleans.

P. S.—Were it not that you have marked your note to me "confidential," I would invite you to use your pleasure as to the insertion of this into a number of your Journal—not that I am desirous to defend my book against criticism, but only in the view of saying what I believe to be quite true—that I have reason to look upon my writings with less doubt as to their usefulness, on account of the undeniable fact that they have met with considerable favor at the hands of the medical public in our country. Yet, after all, perhaps your reviewer may have been pleased to say nothing that I should not be willing to agree to. In that case I should have no answer to make. C. D. M.

